# A new source of root-knot nematode resistance from *Arachis stenosperma* incorporated into allotetraploid peanut (*Arachis hypogaea*)

**DOI:** 10.1038/s41598-019-54183-1

**Published:** 2019-11-27

**Authors:** Carolina Ballén-Taborda, Ye Chu, Peggy Ozias-Akins, Patricia Timper, C. Corley Holbrook, Scott A. Jackson, David J. Bertioli, Soraya C. M. Leal-Bertioli

**Affiliations:** 1 Center for Applied Genetic Technologies and Institute of Plant Breeding, Genetics and Genomics, University of Georgia, Athens, GA United States; 2 Department of Crop and Soil Science and Institute of Plant Breeding, Genetics and Genomics, University of Georgia, Athens, GA United States; 3 Department of Plant Pathology, University of Georgia, Athens, GA United States; 4 Department of Horticulture and Institute of Plant Breeding, Genetics and Genomics, University of Georgia, Tifton, GA United States; 5 USDA-ARS, Tifton, GA United States

**Keywords:** Plant hybridization, Plant breeding, Plant hybridization

## Abstract

Root-knot nematode is a very destructive pathogen, to which most peanut cultivars are highly susceptible. Strong resistance is present in the wild diploid peanut relatives. Previously, QTLs controlling nematode resistance were identified on chromosomes A02, A04 and A09 of *Arachis stenosperma*. Here, to study the inheritance of these resistance alleles within the genetic background of tetraploid peanut, an F_2_ population was developed from a cross between peanut and an induced allotetraploid that incorporated *A. stenosperma*, [*Arachis batizocoi* x *A. stenosperma*]^4×^. This population was genotyped using a SNP array and phenotyped for nematode resistance. QTL analysis allowed us to verify the major-effect QTL on chromosome A02 and a secondary QTL on A09, each contributing to a percentage reduction in nematode multiplication up to 98.2%. These were validated in selected F_2:3_ lines. The genome location of the large-effect QTL on A02 is rich in genes encoding TIR-NBS-LRR protein domains that are involved in plant defenses. We conclude that the strong resistance to RKN, derived from the diploid *A. stenosperma*, is transferrable and expressed in tetraploid peanut. Currently it is being used in breeding programs for introgressing a new source of nematode resistance and to widen the genetic basis of agronomically adapted peanut lines.

## Introduction

*Arachis hypogaea* L. (peanut or groundnut) is an important oilseed, food and forage crop, cultivated worldwide in tropical and subtropical regions with annual production of 64.2 million tons. In the USA, peanut was grown on more than a half million ha (0.72 ha) with an average yield of 4.57 tonnes/ha in 2016 (FAOSTAT 2018). Peanut is an allotetraploid species with very low genetic diversity due to its recent polyploid origin^1–6^. It is highly susceptible to several pests and diseases, including root-knot nematode (RKN) *Meloidogyne arenaria* (Neal). This is in part due to the absence of gene flow with diploid wild relatives with resistant alleles^7^. Root-knot nematode causes substantial yield losses, reduces pod and grain quality, affects plant growth and increases production cost^7,8^. Crop rotation and nematicides are commonly used for nematode management. Due to the ability of *M. arenaria* to infect most crops, few non-host crops are available to reduce populations of *M. arenaria* and crop damage^9,10^. Additionally, chemical control for nematode management is not only costly, but also presents concerns for effects on human health and the environment that have led to the loss of registration of many of the commonly used nematicides^11^. Development of high-yielding and nematode-resistant cultivars is an efficient and effective way to control nematode populations and decrease yield losses while reducing the use of nematicides^12^.

Natural sources of resistance to RKN are not present in cultivated peanut, but found in wild relatives that can be utilized to enhance peanut performance under disease pressure^13–15^. The first use of a wild relative to introgress RKN resistance into peanut dates from 2001, with the release of the cultivar COAN^16^. The resistance was based on a chromosome A09 segment from the wild species *Arachis cardenasii* Krapov. & W.C. Greg.^17^. The segment was introgressed through a backcrossing scheme involving interspecific hybrids^18,19^. Since then, several other cultivars have been released in the U.S. using the same source of resistance: NemaTAM, Tifguard, Webb, Georgia-14N and TifNV-High O/L^20–24^.

Additional sources of resistance are important for the development of new high-yielding and nematode-resistant peanut cultivars, and to reduce the risk of resistance breakdown in the varieties currently used^8^. Previously, we studied the peanut wild relative *Arachis stenosperma* Krapov. & W. C. Greg., which harbors resistance to a number of pests, including the RKN *Meloidogyne* spp. and foliar diseases, such as late leaf spot and rust^25–28^. Subsequently, using diploid mapping populations, we identified a large-effect QTL controlling RKN resistance on chromosome A02 together with minor effect QTLs on A04 and A09 of *A. stenosperma* (accession V10309)^29^. Strong resistance to RKN also has been reported in the wild diploid species *A. batizocoi* Krapov. & W. C. Greg. (accession K9484)^30^.

In this study, an F_2_ population derived from the cross of *A. hypogaea* with an induced allotetraploid (*A. batizocoi* x *A. stenosperma*)^4×^ ^31^ was used to identify genome regions that confer RKN resistance within a tetraploid genetic background; to develop reliable molecular markers tightly linked to the resistance loci for selection in breeding programs; and finally, to characterize the genetic behavior of wild-cultivated crosses. This research will contribute to the production of advanced peanut lines that incorporate wild-derived chromosome segments that confer a new source of resistance to RKN.

## Results

### Nematode screening

**Figure 1:**
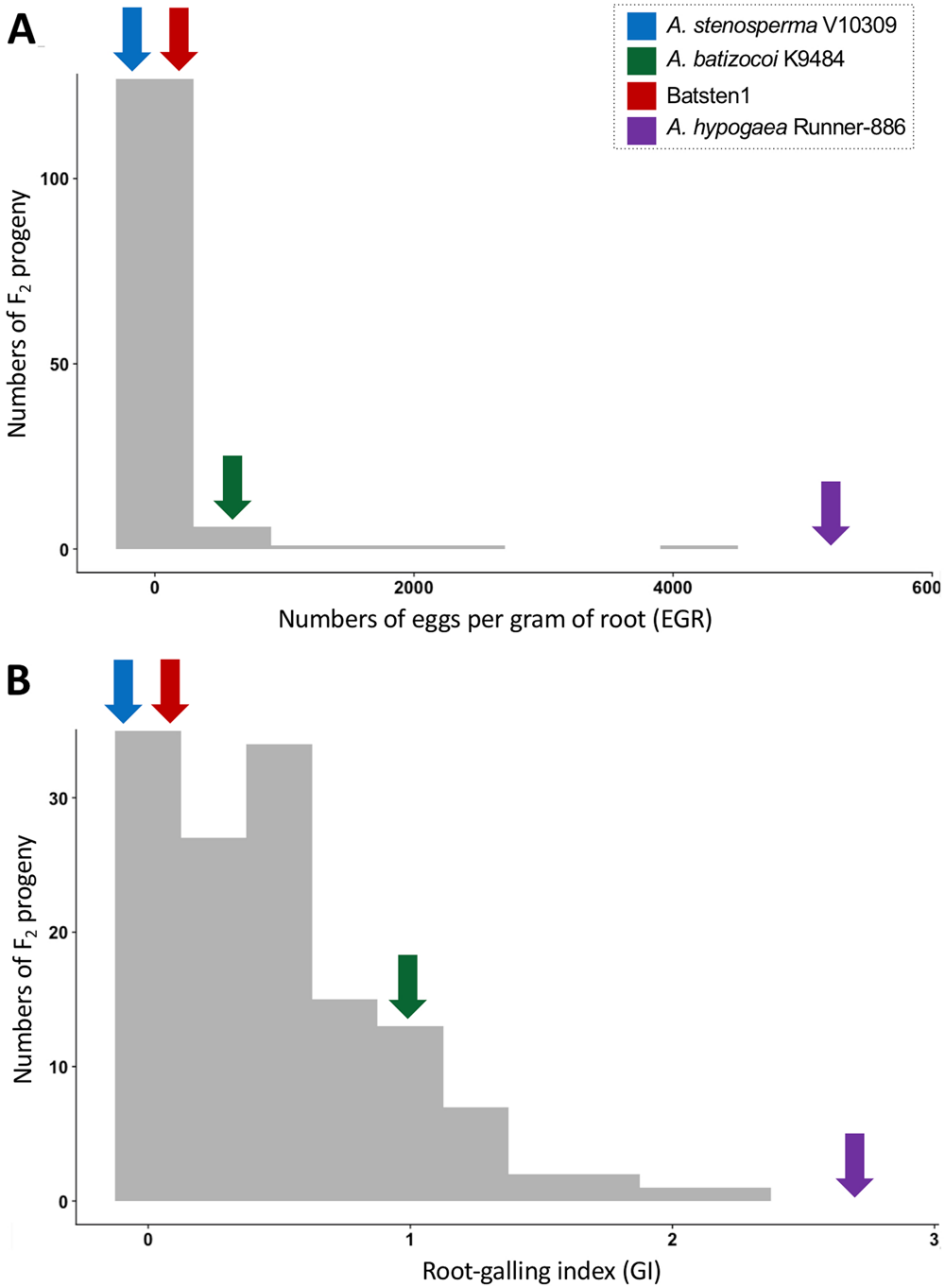
Frequency distribution of disease resistance to *Meloidogyne arenaria* Race 1 among the RBS-F_2_ population for 2014 assay. Eggs per gram of root (EGR) (**A**) and galling index (GI) (**B**). As expected, *A. stenosperma*, BatSten1 and *A. batizocoi* were resistant and *A. hypogaea* Runner IAC-886 was susceptible. The RBS-F_2_ progeny showed a distinctly non-normal distribution, with genotypes having a skewed phenotypic frequency distribution towards resistance (zero value). Number of F_2_ individuals in y-axis and phenotypic values in x-axis. The means of the parents are significantly different (P < 0.05).

Resistance to RKN was evaluated over three years by measuring three traits: EGR (eggs/gram of root), RF (reproductive factor), and GI (galling index). *Arachis stenosperma* V10309*, A. batizocoi* K9484 and the induced allotetraploid BatSten1 ([*A. batizocoi* x *A. stenosperma*]^(2n=4×=40)^ were found to be resistant to RKN, with no or small galls and low egg production. Cultivar Runner-886 was susceptible in all the assays and presented the highest values for all traits (Fig. 1, Table S1 and Tab ‘Traits for F_2_ lines’ in File S1). Individuals from the RBS-F_2_ population (derived from a cross between *A. hypogaea* cv. Runner IAC-886 and the synthetic allotetraploid BatSten1) showed varying degrees of resistance to RKN. The frequency distributions of EGR, RF and GI traits were distinctly non-normal, being skewed towards resistance (Fig. 1). EGR, RF and GI values for the RBS-F_2_ were significantly different at P < 0.05 for 2014 and 2016 assays, but not for 2015. Values for the two parents (Runner-886 and BatSten1) were significantly different (P < 0.05) for 2014 and 2016 assays (in 2015 data for BatSten1 was not available). Transgressive segregation was also observed: across years, on average 49.3%, 48.0% and 59.0% segregating individuals were as, or more, resistant than *A. stenosperma* for EGR, RF and GI, respectively. For the three measured traits, few lines were more susceptible than Runner-886; on average 0.7%, 0.3% and 0.3% individuals were as, or more, susceptible than Runner-886 for EGR, RF and GI, respectively. Several individuals that produced galls did not support the production of nematode eggs. These traits were highly correlated: Pearson correlation was calculated in Minitab v.15.1.0.0 between EGR, RF and GI (Tab ‘Pearson Correlation’ in File S1) and significant values (P $\le$ 0.01) ranged between 0.281 and 0.887.

### Genotyping and genetic mapping

Using the Affymetrix ‘Axiom_Arachis’ SNP array^32,33^ 196 F_2_ progeny and controls were genotyped. Two individuals were eliminated since they had too many missing data points. For the rest of the analyses, data of 194 progenies were used. A total of 1587 polymorphic SNP markers were identified from our filtering strategy, 911 assigned to A-subgenome (*A. stenosperma*-specific markers) and 676 to B/K-subgenome (*A. batizocoi*-specific markers); additionally, 9696 *A. hypogaea*-specific markers were identified. Only markers derived from *A. stenosperma* and *A. batizocoi* were utilized for genetic mapping and QTL identification. The physical positions of the A-genome markers were determined according to the position of their homologues in the *A. duranensis* pseudomolecules and the B-genome markers based on the *A. ipaensis* pseudomolecules (www.peanutbase.org)^2^. After removing low-quality and unlinked markers, 1499 SNP markers were ordered into 20 linkage groups (LGs) that ranged in size from 100.7 cM (LG A05) to 359.5 cM (LG A02), spanning a total map distance of 3984.9. The average distance between adjacent markers ranged from 1.35 cM (LG A01) to 3.8 cM (LG A06) and the largest distance was 29.48 cM (LG A06). The number of markers in each linkage group varied from 36 (LG B04) to 217 (LG A01) (Table S2, Figure S1 and Tab ‘Framework Map’ in File S1). There was a strong relationship between genetic and physical positions of their homologues. As expected, higher recombination frequencies were observed in the distal parts of the chromosomes. LG A07 presents an inversion on the lower end relative to its diploid ancestor, but consistent with the genome sequence of *A. hypogaea* (Figure S2)^1^. The heatmap of the marker-pairwise estimated recombination fractions versus LOD scores indicated that there is only linkage within LGs, with the exception of LGs A05 and B05, where markers seem to be tightly associated with each other, especially at the beginning and end of the linkage groups (Figure S3). This is consistent with tetrasomic genome composition and recombination between alleles between these homeologous chromosomes^1,2^.

### QTL identification

**Figure 2:**
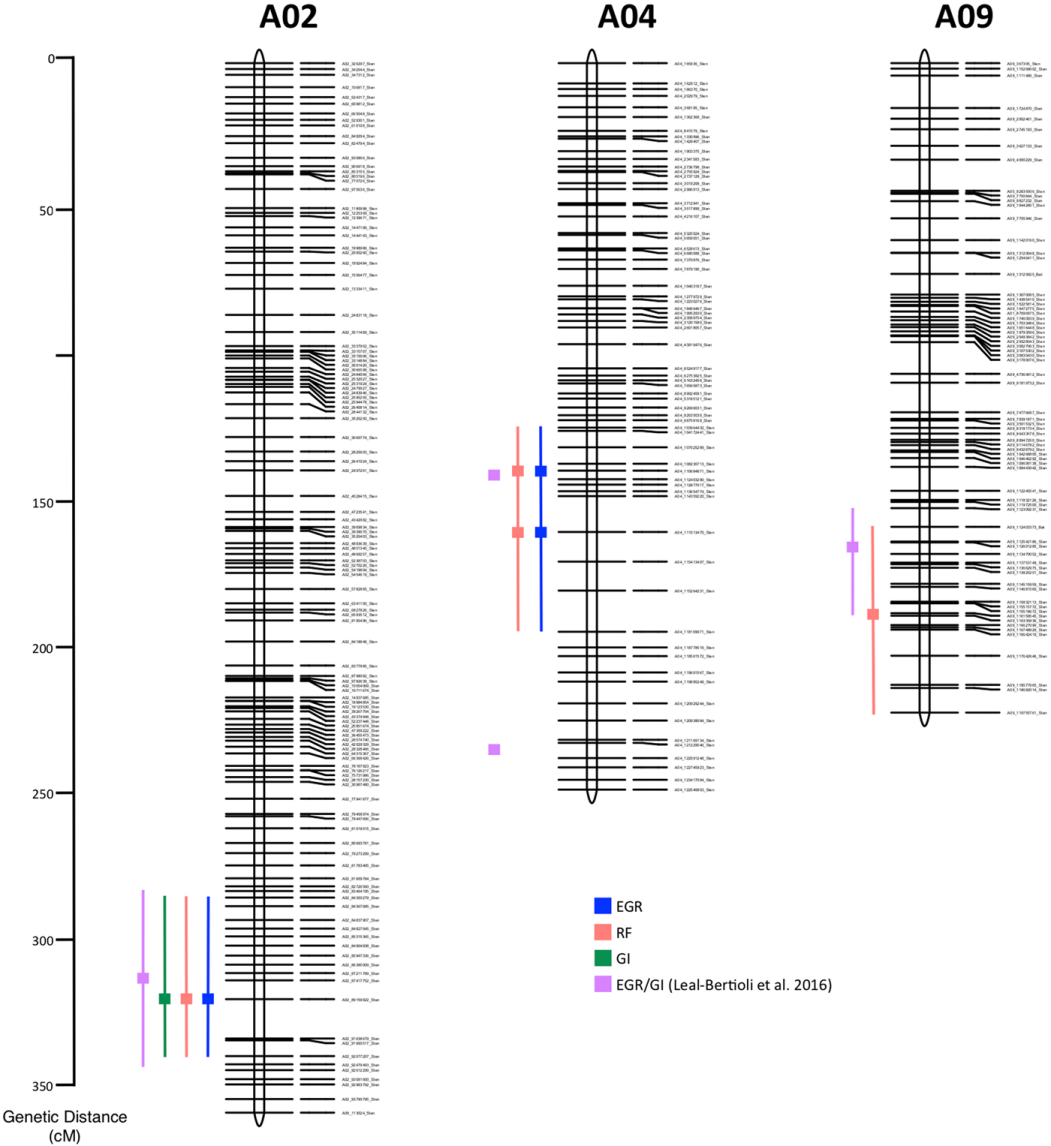
Linkage groups A02, A04 and A09 of the high-density linkage map obtained by the analysis of the RBS-F_2_ population using JoinMap v.4.0. These linkage groups harbored QTL identified in this study (in blue, orange and green) and previously report (purple)^29^. QTL peaks are indicated as colored boxes alongside the linkage groups and QTL intervals as lines.

A large-effect QTL for all three measurements of resistance to RKN was detected at the bottom of LG A02 with LOD scores above 5.9 and with a peak at marker A02_89159922_Sten (320.7 cM) at genome-wise α = 0.05 and 0.01 thresholds (Table 1, Fig. 2, Figure S4A–C and Tab ‘QTL’ in File S1). Using EGR and RF data, the QTL was identified at 1% level of significance (Figure S4A,B and Tab ‘QTL’ in File S1) and for GI data at 5% (Figure S4C and Tab ‘QTL’ in File S1). A second QTL for EGR and RF was observed in LG A04 below the α = 0.05 genome-wise threshold. This QTL seems to comprise two separate loci with LOD scores above 3.2. The highest peak was near A04_111013470_Sten SNP marker (160.6 cM), while the lower peak was near A04_110684871_Sten (139.6 cM) (Table 1, Fig. 2, Figure S5A,B and Tab ‘QTL’ in File S1). A third QTL, for RF, was present in LG A09 with LOD score of 3.7 at marker A09_116335836_Sten (139.6) (Table 1, Fig. 2, Figure S5C and Tab ‘QTL’ in File S1).

**Table 1:** Identified QTL for resistance to RKN on the RBS-F_2_ population.

Trait Symbol	LG^a^	Genetic position^b^	Physical Position^c^	Nearest Marker	LOD^d^	LOD threshold^e^	95% Bayes/LOD interval^f^	Additive Effect^g^	R^2^ (%)^h^	%^i^	QTL Name(s)
EGR2014LOG	A02	320.7	89.2	A02_89159922_Sten	8.1	5.60 (1%), 4.73 (5%)	(320.7)/(314.2–334.1)	0.473	22.2	96.3	A02
RF2014LOG	A02	320.7	89. 2	A02_89159922_Sten	8.3	5.67 (1%), 4.71 (5%)	(320.7)/(314.2–334.1)	0.499	23.0	98.2	A02
GI2014LOG1	A02	320.7	89. 2	A02_89159922_Sten	5.9	6.63 (1%), 5.36 (5%)	(320.7)/(314.2–334.1)	0.065	15.4	61.3	A02
EGR2014LOG	A04	139.6 160.6	110.7 111.0	A04_110684871_Sten A04_111013470_Sten	3.2 3.6	5.60 (1%), 4.73 (5%)	(131.6–160.6)/(125.9–170.7)	−0.005	Do not reduce nematode multiplication		A04a A04b
RF2014LOG	A04	139.6 160.6	110.7 111.0	A04_110684871_Sten A04_111013470_Sten	3.3 3.4	5.67 (1%), 4.71 (5%)	(125.9–160.6)/(124.8–170.7)	−0.068			A04a A04b
RFBLUPS1416LOG	A09	189.3	116.3	A09_116335836_Sten	3.7	5.6 (1%), 4.66 (5%)	(44.7–214.0)/(158.8–222.5)	0.134	9.97	97.8	A09

LOD, logarithm of the odds; EGR2014LOG, Eggs per gram of root Log_10_ transformation for 2014; RF2014LOG, Reproduction factor Log_10_ transformation for 2014; GI2014LOG1, galling index Log_10_ (x + 1) transformation for 2014; BLUPs for Reproduction factor Log_10_ transformation for 2014 + 2016;

^a^Linkage group.

^b^Map position in Kosambi cM.

^c^Physical position is based on *A. duranensis* pseudomolecules (www.peanutbase.org)^2^.

^d^LOD score at QTL peak.

^e^LOD threshold based on 1000 permutations at 1% and 5% level of significance.

^f^95% Bayes credible intervals/LOD support interval.

^g^Positive values indicate that alleles come from *A. stenosperma* V10309 and negative values indicate that alleles come from *A. hypogaea* Runner-886.

^h^Proportion of the phenotypic variance explained by the QTL.

^i^Percentage (%) decrease in nematode multiplication.

All QTLs derived from *A. stenosperma* are colocalized with the ones found in a previous study on a *A. stenosperma*-derived diploid population (Fig. 2 and Tab ‘Colocalization’ in File S1)^29^. The QTL on LG A02 contributed to a percentage reduction up to 98.2% in nematode multiplication. Although putative QTL on A09 did not pass the estimated threshold, it reduced the nematode development up to 97.8%. Putative QTL on A04 did not contribute to improve nematode resistance (Table 1). Although *A. batizocoi* was also found to be resistant to RKN^30^, *A. batizocoi*-derived QTLs associated with nematode resistance were not identified.

**Figure 3:**
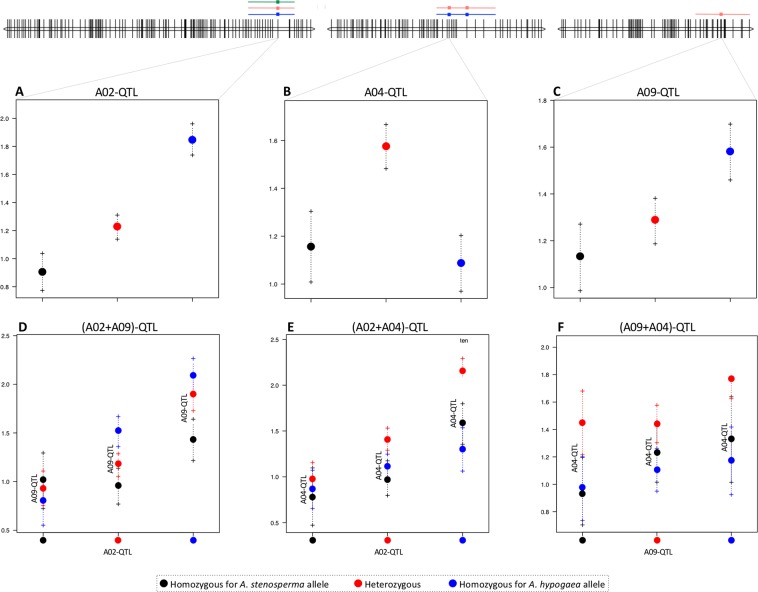
QTL effect plot of Log transformed data number of eggs per gram of root (EGR) at QTL on A02 (A02_89159922_Sten) (**A**), A04 (A04_111013470_Sten or A04_110684871_Sten, both have similar behavior) (**B**) and A09 (A09_116335836_Sten) (**C**); Effect of combination of QTL on A02 and A09 (**D**), A02 and A04 (**E**) and A09 and A04 (**F**); Phenotype values on logarithmic scale (Y-axis) as a function of genotypic class (X-axis). Bars at each genotypic class represent standard error of mean.

The analysis of phenotypic effects of markers tightly linked to QTLs contributing to nematode resistance reveals that most F_2_ lines with *A. stenosperma* alleles at QTLs on LG A02 and A09 showed the lowest phenotypic mean scores for all the measurements of resistance (EGR, RF and GI); whereas, F_2_ plants carrying *A. hypogaea* alleles had a higher incidence of the disease (Fig. 3A,C), Graphs for RF and GI are not shown, but have the same trend. This was also supported with the positive additive effect values (Table 1), suggesting that these genome segments contribute to reduced egg production, nematode reproduction and gall formation, as previously described^29^. This tendency was also observed when analyzing combined effect of two QTLs. F_2_ lines that were homozygous for *A. stenosperma* alleles on A02 and A09 together had low disease scores (Fig. 3D), on average presenting reduction of EGR, RP and GI, of 87.3%, 95.2% and 67.5%, respectively. Conversely, the putative QTL on LG A04 does not produce an improved resistance when the F_2_ lines were homozygous or heterozygous for the *A. stenosperma* alleles (Fig. 3B,E,F), which is supported with the negative additive effect we found for this segment (Table 1). It is possible that this trend on the A04 putative QTL reveals underdominance, where heterozygous F_2_ lines have an inferior performance for nematode infection than either the cultivated or wild homozygous genotypes (Fig. 3B). Alternatively, the lower resistance could have arisen from unfavorable gene interactions^34^.

### Validation of resistance

Progeny of F_2_ lines produced on average 10 seeds per plant (median = 4). Six lines that produced between 12 and 36 seeds were selected to be planted in the field for yield and disease resistance evaluation. Two lines that carried markers associated to QTLs in LG02 and LG09 were selected and advanced to the F_2:3_ generation. To confirm the RKN resistance on the F_2:3_ lines, cuttings where phenotyped for RKN resistance using the same screening approach described in Methods but using eight replicates per genotype and inoculation with 5.1K second-stage juveniles (J2) per pot (Tab ‘Traits for F_2_-derived F_3_ lines’ in File S1). According to Kruskal-Wallis test (P < 0.05), there was a significant difference in RKN resistance between genotypes. Further analysis included the Wilcoxon signed rank test for pairwise comparisons using FDR (false discovery rate) correction, in order to group the samples by significant similarity (P > 0.05). According to the grouping, for EGR, RF and GI all the F_2_-derived F_3_ lines were significantly different from the TifGp-2 susceptible genotype (Table 2). These lines were also genotyped using the ‘Axiom_Arachis v01’ 58 K high-density SNP array^32,33^ and data processed similarly to the F_2_ genotypic data. Only genotyping calls for the *A. stenosperma* and *A. batizocoi*-derived markers were taken into account, the data was then filtered as Batsten1 ≠ *A. hypogaea* (AppendixS2) and then visually inspected for the *A. stenosperma* introgressions along the three chromosome segments associated with nematode resistance in LG A02, A04 and A09. F_2:3_-7 and F_2:3_-34 were found to carry the resistance segments in heterozygous or homozygous states for *A. stenosperma* alleles (Table 2).

**Table 2:** Summary of presence/absence of markers linked RKN resistance segments from *A*.

LG	Marker	F_2_-derived F_3_ lines		*A. hypogaea* TifGP-2
		F_2:3_-7	F_2:3_-34	
A02	A02_83464195_Sten	+/+	+/+	−/−
	A02_84827045_Sten	+/+	−/+	−/−
	A02_85315365_Sten	−/+	−/+	−/−
	A02_86385009_Sten	+/+	−/+	−/−
	A02_89159922_Sten	+/+	−/+	−/−
	A02_91638679_Sten	+/+	−/+	−/−
	A02_92077207_Sten	+/+	−/+	−/−
A04	A04_104172441_Sten	−/+	−/+	−/−
	A04_108230713_Sten	−/+	−/+	−/−
	A04_110684871_Sten	−/+	−/+	−/−
	A04_112403290_Sten	−/+	−/+	−/−
	A04_113654774_Sten	−/+	−/+	−/−
	A04_118561572_Sten	−/+	−/+	−/−
	A04_118778510_Sten	+/+	+/+	−/−
	A04_119895249_Sten	+/+	+/+	−/−
	A04_120929244_Sten	+/+	+/+	−/−
	A04_120938084_Sten	+/+	+/+	−/−
	A04_121169134_Sten	+/+	−/+	−/−
	A04_121229546_Sten	+/+	−/+	−/−
	A04_122540803_Sten	+/+	−/+	−/−
A09	A09_112245541_Sten	−/+	+/+	−/−
	A09_112309231_Sten	−/+	+/+	−/−
	A09_112542186_Sten	−/+	+/+	−/−
	A09_112601285_Sten	−/+	+/+	−/−
	A09_113470052_Sten	−/+	+/+	−/−
	A09_113662975_Sten	−/+	+/+	−/−
	A09_114515959_Sten	−/+	+/+	−/−
	A09_114681560_Sten	−/+	+/+	−/−
	A09_115268602_Sten	−/−	−/−	−/−
	A09_115832113_Sten	−/+	+/+	−/−
	A09_116627090_Sten	−/+	+/+	−/−
	A09_118577665_Sten	−/−	+/+	−/−
	A09_118682014_Sten	−/−	+/+	−/−
Eggs per gram of root - EGR		30.22 ± 28.83(a)	231.82 ± 264.30(a)	2163.78 ± 1688.27(b)
Reproduction factor - RF		0.12 ± 0.08(a)	0.21 ± 0.23(a)	5.49 ± 2.96(b)
Galling index - GI		0.17 ± 0.41(a)	0.00 ± 0.00(a)	2.57 ± 0.79(b)

*stenosperma* in chromosomes A02, A04 and A09, and EGR, RF and GI disease average values and grouping by Wilcoxon signed rank (P > 0.05) for selected RBS-F_2:3_ lines and susceptible control. Homozygous for *A. hypogaea* alleles as “−/−“; homozygous for resistance segments as “+/+”; and heterozygous as “−/+”.

### Inheritance patterns and segregation distortion

**Figure 4:**
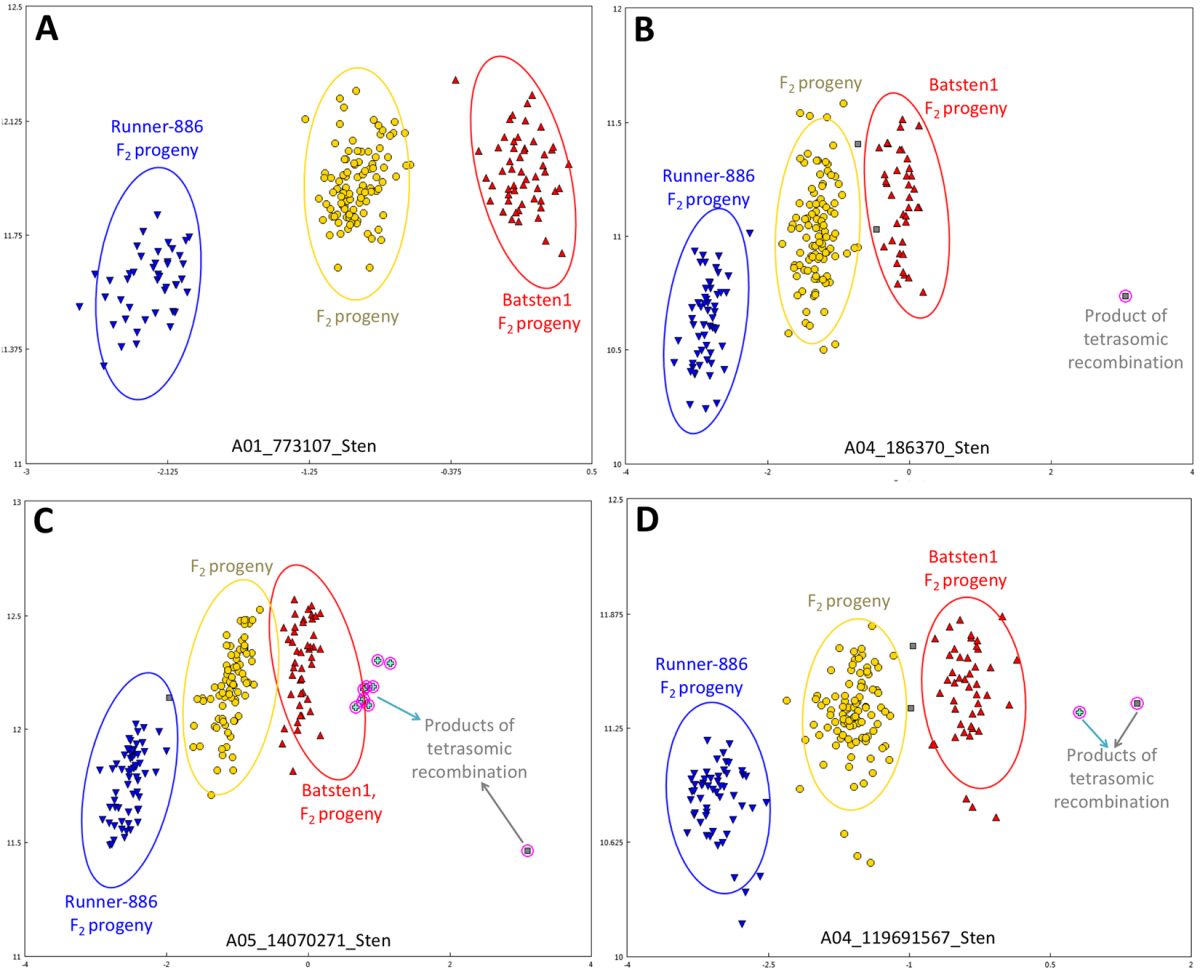
Examples of SNP markers segregation in the F_2_ population under disomic (**A**) and tetrasomic inheritance (**B**–**D**). One product of homeologous recombination is detected and labeled as gray (triplex/quadriplex) (**B**) and two products of tetrasomic recombination are detected and labeled as green (triplex) and gray (quadriplex) (**C**,**D**).

In an F_2_ population with disomic recombination, three main genotypic classes are expected: the two parental and the hybrid types (Fig. 4A). A set of 1156 SNPs (77.1%) were inherited as expected under disomic inheritance: 694 (46.3%) markers followed the Mendelian segregation ratio expected for an F_2_ population (1:2:1) and 462 (30.8%) showed significant deviation (P < 0.01) (Table 3, Tab ‘Tetrasomic Recombination’ in File S1). From the 234 (15.6%) distorted loci located in the A-genome, distorted blocks were skewed toward the cultivated genotype specially on LGs A04 and A05. Among the 228 (15.2%) loci in B/K genome, we found a biased segregation in favor of wild in B06 and an excess of cultivated alleles in B07 (Figure S6 and Tab ‘Tetrasomic Recombination’ in File S1).

**Table 3:** Segregation of SNP markers in the RBS-F_2_ population.

Type of inheritance						
Disomic				Tetrasomic		Total
A-genome		B/K-genome		A-genome	B/K-genome	
Mendelian	Distorted	Mendelian	Distorted	238 (15.9%)	105 (7.0%)	1499
394 (26.3%)	234 (15.6%)	300 (20.0%)	228 (15.2%)			

*Percentage of marker in each category is indicated in parenthesis.

**Figure 5:**
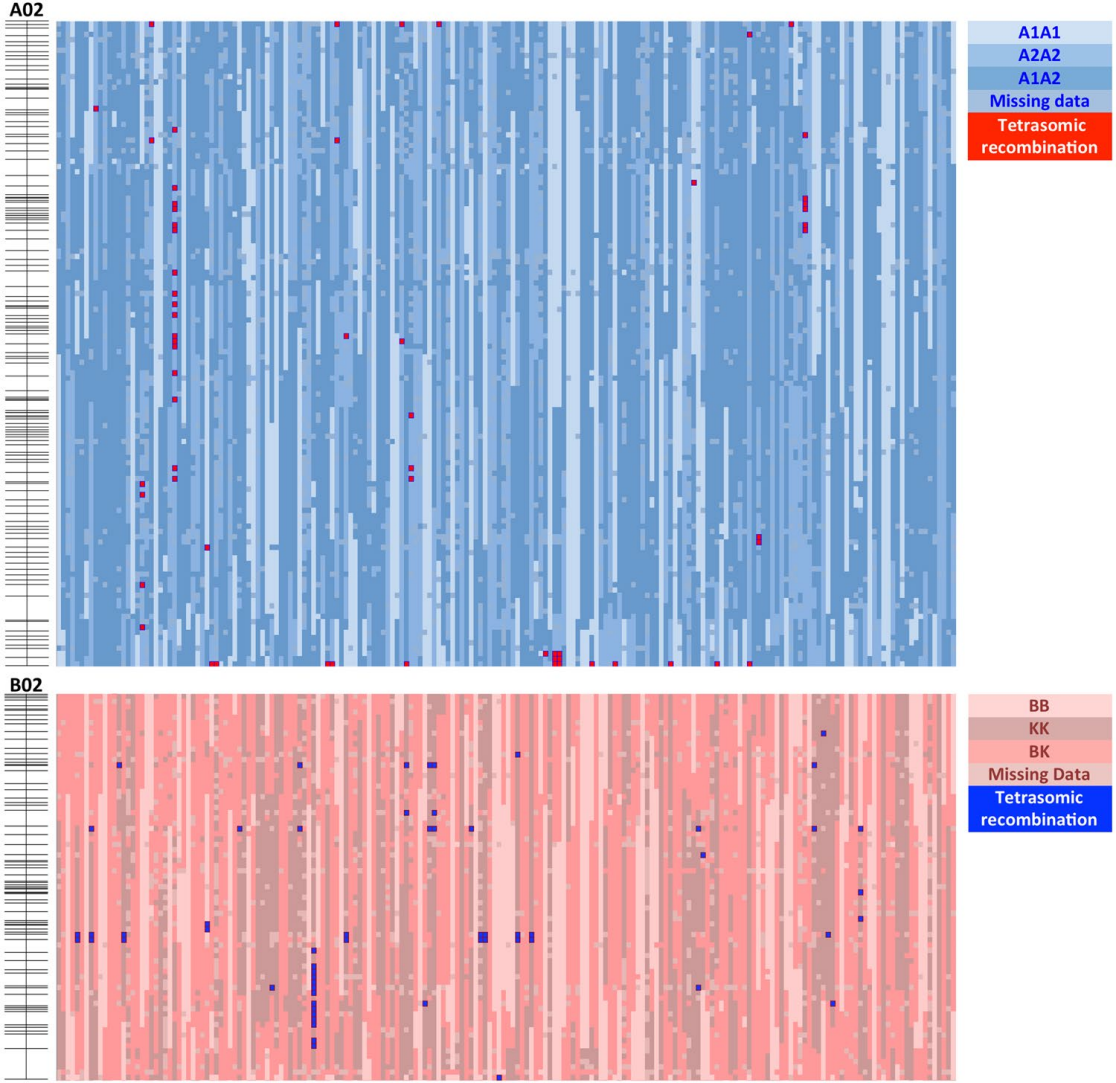
Genotyping color map of 194 F_2_ progeny for linkage groups A02 and B02. Each column represents an F_2_ line and rows represent markers. Blue and red colors denote the A and B/K-subgenomes, respectively. Cultivated (A1A1), wild (A2A2) and heterozygous (A1A2) genotypes for the A-genome are represented by light blue, blue and dark blue, respectively. Cultivated (BB), wild (KK) heterozygous (BK) genotypes for the B-genome are represented by light red, dark red and red, respectively. Red in the A-genome and blue in the B-genome color indicate tetrasomic recombination events. Linkage maps on the side are included for illustration purposes. See more linkage groups in the tetrasomic color map (Tab ‘Tetrasomic Recombination’ in File S1).

During marker analyses, it was also noted that several markers exhibited unexpected genotypic classes, in other words additional clusters to the three genotypic classes expected with disomic recombination. To investigate the nature of these markers, the clustering of 1499 polymorphic SNPs for the 194 F_2_ lines were visually inspected and confirmed. The percentage of markers in each LG that showed tetrasomic recombination in at least one genotype, ranged from 4.3% (LG B03) to 75% (LG A04) with an average of 22.9% (343 SNPs) (Table S2). These informative SNPs showed unexpected clustering patterns (Fig. 4B–D), which was explained by recombination between homeologous chromosomes (Table 3, Tab ‘Tetrasomic Recombination’ in File S1). A total of 565 individual data points across the whole data set (1499 SNPs x 194 individuals) (0.19%) were informative to identify homeologous recombination. Lines that are triplex (single allele replaced by its homeolog) or quadriplex (both alleles replaced by homeolog) are expected only when individuals have undergone polysomic recombination; Fig. 4C,D shows examples of assays that can distinguish two products of homeologous recombination: triplex (green) and quadriplex (gray). In the example in Fig. 4B. It is unclear whether or not the individual that has undergone homeologous recombination for this locus is triplex or quadriplex, since there is no information about the allele dosage in the B-subgenome. Segregation ratio and distortion was not possible to describe for the markers with tetrasomic behavior, since the information about the F_1_ hybrid and its gametic allelic constitution is unknown. Additionally, we observed blocks of genome substitution especially on LG A02/B02 (Fig. 5), A03, A04, A08 and A10/B10. A04 was the LG with highest number of markers exhibiting homeologous recombination (Tab ‘Tetrasomic Recombination’ in File S1). All of above is consistent with previous reports for F_2_ progeny^35^, RIL populations^32,36^ and when mapping *A. hypogaea* RIL sequences against *A. duranensis* and *A. ipaensis* genomes^2^.

## Discussion

The peanut wild relative *A. stenosperma* has been shown to be resistant to multiple pests and pathogens including *M. arenaria* Race 1. This resistance is manifest at multiple stages of the infection cycle: both much lower rates of penetration of nematodes, and hypersensitive response to the few nematodes that do penetrate are observed^27^. Genes involved in hypersensitive response and secondary metabolite production for defense against nematode infection have been identified in differential analyses of gene expression and histology^27,28,37^. Prior to the present work, RKN resistance segments on A02, A04 and A09 of the wild *A. stenosperma* were discovered using the simplified genetic context of a diploid mapping population derived from a cross of *A. duranensis* x *A. stenosperma*^29^. Here, we used an F_2_ population from the cross of the cultivar Runner-886 and the wild-derived allotetraploid Batsten1^31^ to analyze the genome regions conferring resistance in a tetraploid background. The aim of this work was to provide a framework of knowledge to incorporate this new source of resistance into elite peanut cultivars through marker-assisted backcrossing schemes (MABC)^38^, work that is now in progress. Using the high-density genetic map and RKN phenotypic data measured on the F_2_ progeny, allowed us to validate the previously described diploid QTLs, in the tetraploid context of cultivated peanut. We identified three *A. stenosperma* QTLs associated with nematode resistance: on the bottom of LG 02, middle of LG 04 and bottom of LG 09.

The QTL on LG A02 was consistently associated with lower EGR, RF and GI (measures of resistance) (Table 1, Fig. 2, Figure S4, and Tab ‘QTL’ in File S1). The QTL located on LG A02 was found in the same region as the previously described diploid QTL (Fig. 2 and Tab ‘Colocalization’ in File S1). The diploid QTL peak was mapped at 87.4Mbp with an interval from 83.6Mbp to 92.5Mbp^2,29^. Here in the tetraploid context we located this resistance segment at 89.2Mbp with an interval between 84.3Mbp and 92.1Mbp. The second tetraploid QTL on LG A04 was near the diploid QTL at 111.0 Mbp. The QTL we identified in A09 was located at 116.3 Mbp, a little further from the previously reported diploid position (112.8 Mbp) (Fig. 2 and Tab ‘Colocalization’ in File S1)^2^. With this information, we could demonstrate that RKN resistance from *A. stenosperma* is transferable and stable in tetraploid genotypes.

The chromosome segment conferring resistance on LG A02 is homologous to the region of the reference genome of *A. duranensis*, that harbors multiple resistance-genes (R-genes)^2^. A gene encoding a toll/interleukin-1 receptor (TIR)-like-nucleotide binding (NBS)-leucine-rich repeat (LRR), associated with plant immune defenses^39–41^ is close to the A02-QTL peak (89.2Mbp) (Fig. 2 and Tab ‘Colocalization’ in File S1). Recently, at a different genome location, a different TIR-NBS-LRR gene was observed to be constitutively expressed in the cultivar Tifguard, which carries RKN resistance from the wild species *A. cardenasii* and absent in the susceptible cultivar Gregory^42^.

The effects of the A02 and A09-QTL on nematode resistance was confirmed in selected F_3_ lines (F_2:3_-7 and F_2:3_-34) carrying resistance loci in a heterozygous (Table 2, “−/+”) or homozygous (Table 2, “+/+”) state. The presence of *A. stenosperma* alleles at the QTL positions significantly reduced egg production (EGR), inhibited the nematode reproduction (RF) and decreased gall formation in comparison with susceptible genotype TifGP-2. We can infer that the presence of resistance segments is important to halt the completion of different steps of nematode life cycle. In the future we intend to carry out histological work on advanced backcross lines carrying different combinations of wild species chromosome segments, to provide insights into the timing and nature of the resistances conferred by these chromosome segments as in Proite *et al*.^27^. Currently lines F_2:3_-7 and F_2:3_-34 are being crossed and backcrossed with several agronomically elite peanut lines.

Although the main focus of this study was to discover, introgress and validate QTLs associated with nematode resistance, interesting non-disomic inheritance of markers was also detected in the F_2_ genotyping data. In a tetraploid hybrid context with cultivated peanut, the *A. stenosperma* genome is expected to recombine predominantly with *A. hypogaea* A-subgenome and the K genome (B genome *sensu lato*) of *A. batizocoi* with the *A. hypogaea* B-subgenome. Previously, genetic mapping and QTL identification studies assumed disomic inheritance in *Arachis* tetraploid species^43–47^. However, Leal-Bertioli *et al*.^36^ provided the first molecular evidence of non-homologous alleles recombination in peanut, when unexpected genotyping patterns where detected for some loci in cultivated x artificially induced allotetraploid RIL lines as well as the parent. Later, this phenomenon was reported in an F_2_ progeny, also derived from a peanut by synthetic tetraploid cross^35^. More recently tetrasomic recombination has been reported in pure *A. hypogaea* crosses^1,2,32^.

In this study, using Affymetrix genotyping, most SNP markers were inherited as expected for disomic segregation, but others appeared to have undergone homeologous recombination. The percentage of markers showing tetrasomic recombination found in this study (22.9%) was higher than previously found by other groups^35^ for an F_2_ progeny (11.05%). LG A04 had the highest number of markers showing tetrasomic behavior (Table S2); in one F_2_ plant we observed almost a complete substitution of the B/K04 and B/K10 alleles by their homeologs on A04 and A10 (Tab ‘Tetrasomic Recombination’ in File S1). This recombination between homeologous chromosomes supports the idea that cultivated peanut has segmental genetic inheritance, where behavior is mostly disomic but partially polysomic. Since tetrasomic recombination occurs mostly in gene-rich regions (distal parts of chromosomes), understanding this phenomenon is not only important to avoid disregarding these genomic segments during genetic mapping^32^, but also to address peanut breeding strategies to transfer genes between species and to accelerate the accumulation of favorable alleles through marker-assisted introgression^35^.

From the analysis of segregation distortion (Chi-square test, P < 0.01), we observed that markers with significant deviation from expected Mendelian segregation ratio (1:2:1) were distributed as clusters, possibly located within segregation distortion regions (SDRs)^48^. The SDRs have been shown to be present in interspecific or wide crosses in plants^48–51^ and also described in *A. hypogaea* intra and interspecific mapping populations^35,44,46,52^. Here, distorted markers on LG A04, A05 and B07 showed a bias toward *A. hypogaea*, indicating strong selection against the *A. stenosperma* alleles. In chromosome B06, we found a biased segregation in favor of wild *A. batizocoi* (FigureS 6 and Tab ‘Tetrasomic Recombination’ in File S1). In general, more distorted loci were found in favor of the *A. hypogaea* parent.

## Materials and Methods

### Plant materials

Wild *Arachis* accessions were obtained from the USDA-GRIN system (https://www.ars-grin.gov/). To introgress the diploid nematode resistance into tetraploid peanut, a synthetic allotetraploid (BatSten1) was created using the peanut wild relatives *A. stenosperma* PI666100 (original collection voucher V10309) and *A. batizocoi* PI298639 (original collection voucher K9484), as described in Leal-Bertioli *et al*.^36^. This wild-derived allotetraploid combines the A genome of *A. stenosperma*^6,53^ and a K genome (B genome *sensu lato*) of *A. batizocoi*^6,54^. An F_2_ segregating population was created by selfing an F_1_ derived from a cross between *A. hypogaea* cv. Runner IAC-886 (herein called Runner-886) and BatSten1. This population was named RBS-F_2_ and had initially, 196 individuals. To be able to perform the bioassays in different years, the RBS-F_2_ population was maintained in the greenhouse by vegetative propagation. The majority of the individuals, but not all, were maintained for the duration of this work.

### Root-knot nematode resistance evaluation

The RBS-F_2_ population was evaluated for resistance to root-knot nematode (RKN) *M. arenaria* Chitwood race 1 under greenhouse conditions^29,55^ in a randomized complete block design with five replicates per genotype. The tetraploid parents (BatSten1 and Runner-886) and the diploid wild species *A. batizocoi* and *A. stenosperma* were used as controls. RKN populations were maintained and extracted from eggplant (*Solanum melongena* cv. Black Beauty). *Arachis* seeds were planted in nursery pots (15 cm in diameter 10 cm in height) filled with Promix (Premier Horticulture, Quakertown, PA) and maintained in a greenhouse. Two-month-old cuttings from each F_2_ line were established in steam-sterilized sandy soil and inoculated with 10,000 second-stage RKN juveniles (J2) by distributing the inoculum in two 2-cm deep holes at the base of the plant. Eight weeks later, plants were uprooted, rinsed, and the roots weighed after removing excess water with a paper towel. Roots were stained with 0.05% phloxin B solution for 3 to 5 min. Nematode eggs were extracted from roots using 0.5% NaOCl^56,57^. Assays were conducted over three years, with 155, 105 and 99 segregating F_2_s, respectively, as a few individuals died with time. Resistance was assessed using three different traits: 1) Numbers of eggs per gram of root (EGR); 2) Nematode reproductive factor (RF = Pf/Pi; where Pf is the final egg population and Pi the initial J2 population^58^; and 3) Root-galling index or egg masses: 0 (no galling or no egg masses), 1 (1–2 galls), 2 (3–10 galls), 3 (11–30 galls), 4 (31–100 galls) and 5 (more than 100 galls or egg masses per root system)^57^.

### Statistical analysis

The Shapiro-Wilk test was used to test normality of phenotypic data. Non-parametric Kruskal-Wallis one-way analysis of variance^59^ was used to assess the global differences in phenotypic traits at a 5% level of significance (P < 0.05) among RBS-F_2_ lines and controls for each year using the Statistical package R. Non-normal phenotype data were transformed to Log_10_ and Log_10_(x + 1) for QTL identification. Additionally, the Best Linear Unbiased Predictors (BLUPs) of random effects were calculated for each trait using the *ranef* function in R. BLUPs were calculated in order to control for missing phenotypic data and transformed to Log_10_ and Log_10_(x + 1).

### SNP genotyping, analysis and data filtering

Genomic DNAs of 196 individuals from the RBS-F_2_ population and controls (BatSten1, Runner-886, *A. stenosperma* and *A. batizocoi*) were extracted from leaves using the DNeasy *Plant* Mini *Kit* (*QIAGEN*) according to manufacturer’s instructions. DNAs were quantified with PicoGreen and samples were submitted for genotyping with the ‘Axiom_Arachis v01’ 58 K high-density SNP array^32,33^. The genotypic data were extracted, processed and analyzed using the Axiom Analysis Suite 2.0 software (http://www.affymetrix.com). Output was analyzed using Unix scripts (AppendixS1) and data were visualized as a color map in Microsoft Excel (Tab ‘Framework Map’ in File S1). The strategy to identify polymorphic SNP markers included three different steps:

Firstly, informative SNP assay results were extracted from SNP calling using a panel of diploid species plus a single tetraploid genotype (*A. hypogaea* Runner-886). This set of markers was filtered to reveal SNP markers specific to each of the three parental species in the pedigree of the F_2_ population as follows:

- *A. stenosperma-*characteristic markers: *A. stenosperma* ≠ (*A. batizocoi* = *A. hypogaea*)
- *A. batizocoi*-characteristic markers: *A. batizocoi* ≠ (*A. stenosperma* = *A. hypogaea*)
- Runner-886 characteristic markers: *A. hypogaea* ≠ (*A. stenosperma* = *A. batizocoi*)

Secondly, SNP assay results were extracted from SNP calling of tetraploid genotypes only (Runner-886, BatSten1 and RBS-F_2_ population). Finally, the three sets of informative SNP markers identified in the first step were retrieved using the panel of tetraploid genotypes, followed by merging and filtering as Batsten1 ≠ *A. hypogaea*.

### Genetic Mapping and QTL discovery

Genetic maps for A and B subgenomes were constructed using Kosambi’s genetic map function^60^ and maximum likelihood algorithm in JoinMap v.4.0^61,62^. The goodness of fit Chi-square test was performed to evaluate the expected 1:2:1 segregation ratio for the F_2_ population for each locus (P < 0.01). The genetic map was visualized by calculating pairwise logarithm of the odds (LOD) scores and recombination fractions using the *plot.rf* function in R/QTL^63,64^.

The genetic map in combination with transformed RKN measurements of resistance for three years were used for QTL identification using R/QTL software following the procedure described in “A guide to QTL mapping with R/qtl”^63^. Due to the spike observed in the phenotype data distribution at zero (*null* phenotype, Fig. 1), we employed a two-part binary plus normal analysis method using the *scanone* function^63–65^. The two-part model is suitable for data with non-normal distribution prior and after transformations, as we observed in our case (Fig. 1). This model performs two different analyses. First, the phenotype is treated as a binary trait (0 or >0), and then as a quantitative trait, for those individuals with phenotypic values above zero^65,66^. The two-part model calculates the LOD scores for each tested genome position to assess the significant association with the trait of interest^63–65^. 1000 permutations were used to identify genome-wide LOD significance thresholds for QTL identification at 1% and 5% level of significance^67–69^. 95% Bayesian credible interval was calculated with the *bayesint* function and LOD support interval with *lodint function* in R/qtl. The percentage of phenotypic variability explained by a QTL (R^2^) and the estimated effect was assessed using the *fitqtl* function in R/qtl^63^. Physical positions for each marker on the A and B subgenomes were determined, respectively, based on the *A. duranensis* and *A. ipaensis* pseudomolecules (www.peanutbase.org)^2^.

### Meiotic behavior analysis and tetrasomic inheritance

Given that the genome of tetraploid peanut harbors regions where tetrasomic recombination can occur^1,2,32,35,36^, we examined our data for evidence of such chromosomal behavior. When assuming recombination only between homologous chromosomes, the expected segregation ratio in the F_2_ population should be 1(A_1_A_1_):2(A_1_A_2_):1(A_2_A_2_) for A-subgenome and 1(BB):2(BK):1(KK) for the B/K-subgenome, but when homeologous chromosomes recombine during the parental and/or F_1_ meiosis, the expected segregation ratio in the F_2_ progeny changes, as described by Nguepjop *et al*.^35^.

In order to determine lines exhibiting tetrasomic recombination and the markers associated with these regions, all 1499 polymorphic markers were visually and manually inspected. The criterion was to analyze data points with “No Call” data or forming unexpected genetic clusters, similar to the rationale described in Leal-Bertioli *et al*.^36^ but applied to an F_2_ population. Genotypes and markers were scored as “tetrasomic” on the color map (Tab ‘Tetrasomic Recombination’ in File S1, red on A-subgenome and blue in B-subgenome). Additionally, segregation distortion from 1:2:1 ratio (P < 0.01) was analyzed for markers segregating in a disomic manner.

## Conclusions

This research allowed us to transfer to and validate QTLs for RKN resistance derived from the diploid *A. stenosperma* into a tetraploid background. We observed that the chromosome segments carrying RKN resistance behaved normally in an induced tetraploid and in crosses with cultivated peanut. Currently, diagnostic markers are being used for the selection of backcrossed lines with resistance to RKN. Additionally, we were able to confirm the segmental genetic behavior with predominantly disomic, but partly tetrasomic genetic inheritance. This research will contribute to the production of peanut varieties that incorporate a new source of resistance to RKN from the wild species *A. stenosperma*. Since all the current RKN resistant cultivars have alleles derived *A. cardenasii*, expanding the gene pool will help ensure continued protection of the peanut crop from losses due to this pest. It will also enable lower inputs of agrochemicals and fuel, reducing environmental impact, higher profitability and more stable peanut yields.

## Supplementary information

FileS1

AppendixS1

AppendixS2

Supplementary File
