# Supplementary material for: A new source of root-knot nematode resistance from Arachis stenosperma incorporated into allotetraploid peanut (Arachis hypogaea)
Source: Sci Rep. 2019 Nov 27;9:17702. doi: 10.1038/s41598-019-54183-1 (PMC6881346; doi:10.1038/s41598-019-54183-1)
Supplement: Supplementary file 4 — Supplementary File [file 41598_2019_54183_MOESM4_ESM.pdf]

A new source of root-knot nematode resistance from *Arachis stenosperma* incorporated into allotetraploid peanut (*Arachis hypogaea*)

Carolina Ballén-Taborda, Ye Chu, Peggy Ozias-Akins, Patricia Timper, C. Corley Holbrook, Scott A. Jackson, David J. Bertioli and Soraya C.M. Leal-Bertioli

Table S1: Root-knot nematode phenotyping values for control genotypes for 2014, 2015 and 2016 and number of phenotyped individuals in comparison with midparent, *A. stenosperma* and *A. hypogaea* Runner-886. Number of eggs per gram of root (EGR), Reproduction factor (RF) and galling index (GI).

	EGR-2014	EGR-2015	EGR-2016	RF-2014	RF2015	RF-2016	GI-2014	GI-2015	GI-2016
A. stenosperma V10309	0	250 (±433.01)	13.75 (±27.5)	0	0.06 (±0.09)	0.015 (±0.03)	0	0.5 (±0.58)	0
A. batizocoi K9484	621.09 (±768.27)	26.09 (±58.33)	88.33 (±152.99)	0.04 (±0.042)	0.033 (±0.074)	0.06 (±0.104)	1 (±1.41)	0.2 (±0.45)	1 (±1.73)
BatSten1	2.70 (±6.04)	-	55.67 (±96.42)	0.001 (±0.002)	-	0.1 (± 0.17)	0	-	0.33 (±0.58)
Runner-886	5320.67 (±5686.27)	3613.48 (±2518.27)	1971.2 (±2046.31)	1.24 (±1.35)	5.85 (±4.85)	4.49 (±5.89)	2.75 (±2.22)	2 (±1.15)	2.4 (±0.55)
Total phenotyped individuals	155	99	99	155	98	99	155	105	99
MidParent	2661.69	-	1013.43	0.617	-	2.294	1.375	-	1.37
More resistant than midparent	154	-	92	152	-	99	149	-	98
Less resistant than midparent	1	-	7	3	-	0	6	-	1
Equal or more resistant than A. stenosperma V10309	24	94	30	24	78	42	40	97	40
Less resistant than A. stenosperma V10309	130	6	71	130	21	59	115	9	61
Equal or more susceptible than A. hypogaea IAC886	0	0	2	1	0	0	0	1	0

Table S2: Description of 10 linkage groups in the calculated genetic map. Genetic length in cM, number of SNPs in each LG, average and largest distance between SNP markers and number of SNP markers that undergone tetrasomic recombination. Lowest and highest values in each category are highlighted in red.

LG[a]	Length (cM)	SNP number	Average distance (cM)[b]	Largest distance (cM)[c]	Tetrasomic markers (%)[d]
A01	290.56	**217**	**1.35**	8.26	12.0
A02	**359.52**	122	2.97	13.48	28.7
A03	242.26	72	3.41	17.46	31.9
A04	248.87	68	3.71	14.12	**75.0**
A05	**100.69**	38	2.72	**8.04**	10.5
A06	315.52	84	**3.80**	**29.48**	20.2
A07	167.50	61	2.79	13.74	23.0
A08	203.99	81	2.55	12.80	32.1
A09	222.49	71	3.18	11.11	15.5
A10	143.99	52	2.82	9.55	59.6
B01	191.36	92	2.10	13.58	12.0
B02	193.57	73	2.69	15.41	37.0
B03	232.70	70	3.37	14.19	**4.3**
B04	113.80	**36**	3.25	11.41	13.9
B05	112.27	44	2.61	9.81	22.7
B06	176.56	70	2.56	8.27	12.9
B07	139.03	57	2.48	16.36	5.3
B08	178.78	73	2.48	14.55	16.4
B09	171.40	61	2.86	13.71	16.4
B10	180.02	57	3.21	24.37	26.3
Total/average	Total = 3984.89	Total = 1944	Average = 2.85	-	Average = 22.9

[a] Linkage group
[b] Average distance between SNP markers (cM)
[c] Largest distance between SNP markers (cM)
[d] Percentage (%) of markers showing tetrasomic recombination (%)

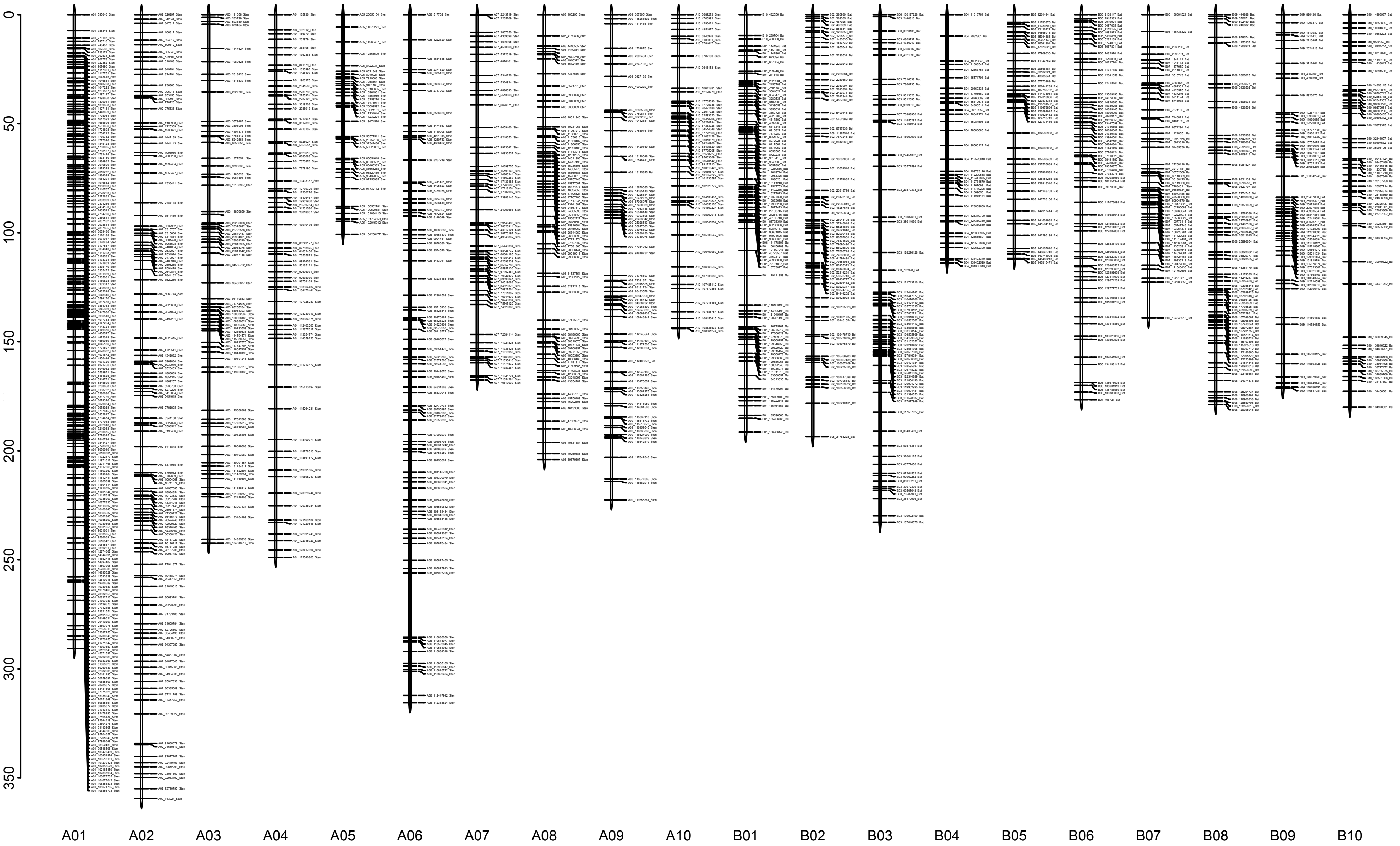

Figure S1: High-density linkage map obtained through the analysis of the RBS-F_2 population using JoinMap. 20 linkage groups for A subgenome and B subgenome. Linkage group names are shown at the bottom of the figure; distance (cM) is shown to the left of the figure and marker names are shown to the right of each LG.

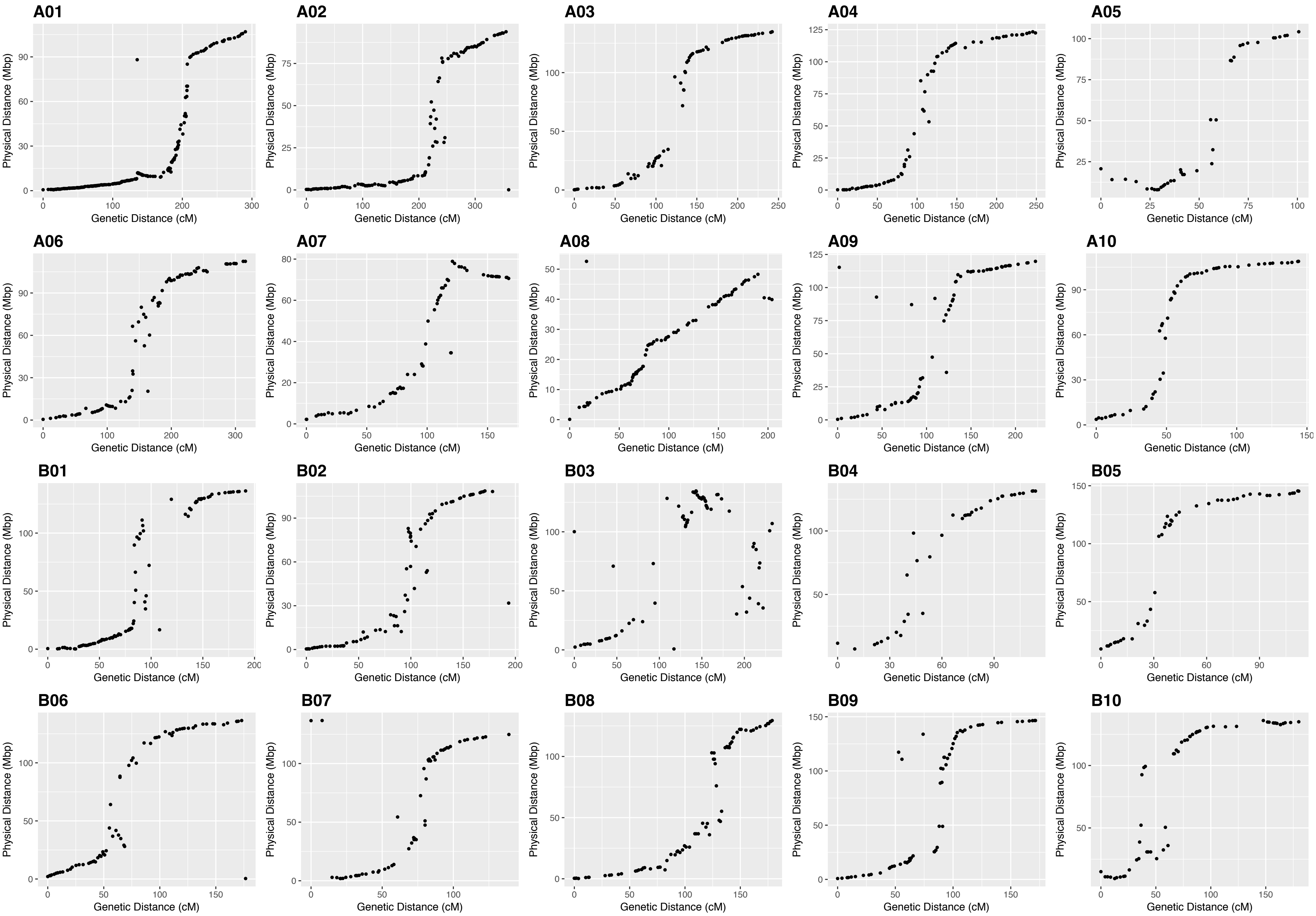

Figure S2: Relationship plot between genetic distance (x-axis) and physical distance (y-axis) for 10 A-subgenome (markers derived from *A. stenosperma*) and 10 B/K-subgenome (markers derived from *A. batizocoi*) linkage groups.

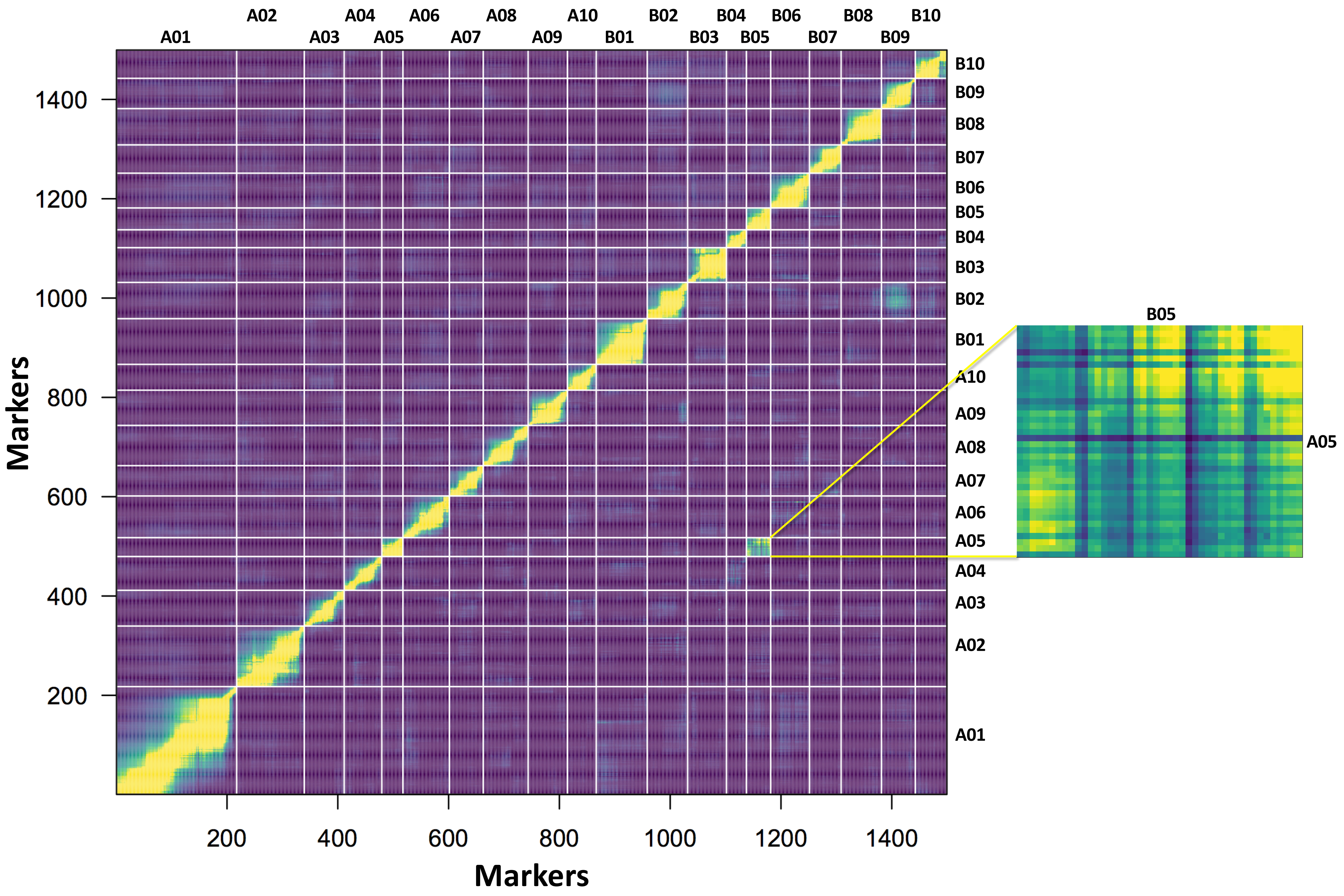

Figure S3: Plot of estimated recombination fractions (above diagonal) and LOD scores for tests of r = 1/2 (below diagonal) for all pairs of markers in the linkage map. Yellow indicates linkage, while blue indicates pairs that are not linked. Magnified look of the relationship between homeologous chromosomes A05 and B05 is shown in the right panel.

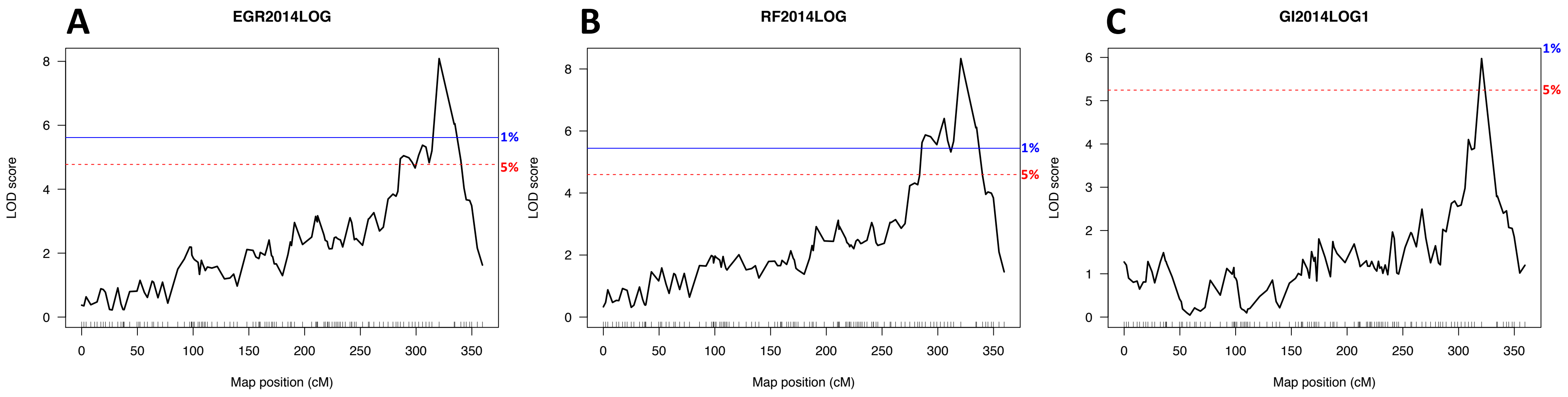

Figure S4: Identified QTL on chromosomes A02 using two-part model for RKN resistance for EGR2014LOG, Eggs per gram of root Log10 transformation for 2014 **(A)**; RF2014LOG, Reproduction factor Log10 transformation for 2014 **(B)**; and GI2014LOG1, galling index Log10 (x+1) transformation for 2014 **(C)**. Genome-wise LOD threshold at 1% (P<0.01, horizontal blue solid lines) and 5% (P<0.05, horizontal red dashed lines) level of significance based on 1000 permutations.

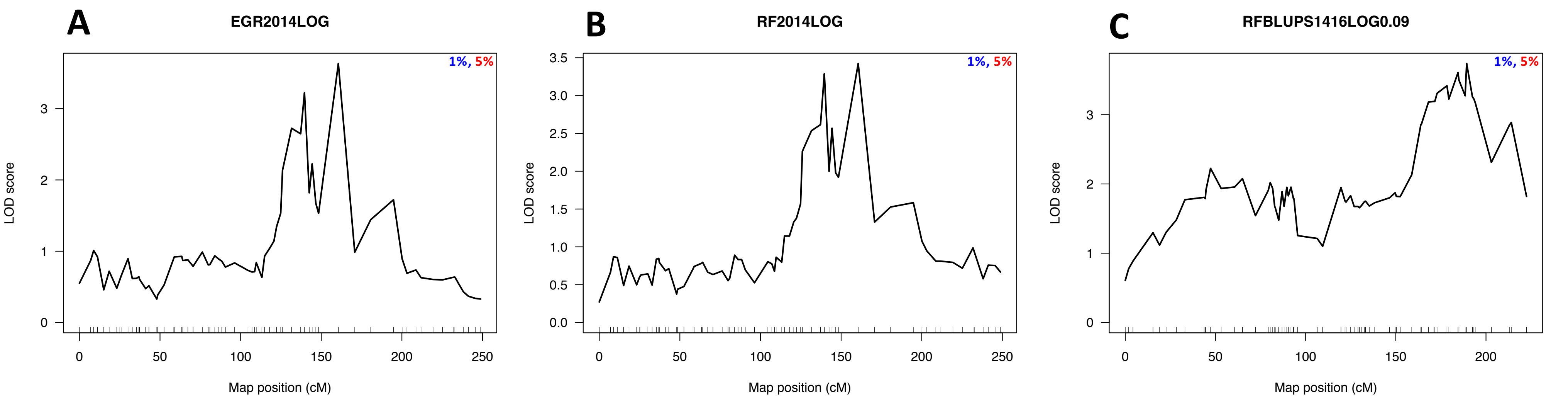

Figure S5: Identified QTL on chromosomes A04 **(A-B)** and A09 **(C)** using two-part model for RKN resistance for EGR2014LOG, Eggs per gram of root Log10 transformation for 2014 **(A)**, RF2014LOG, Reproduction factor Log10 transformation for 2014 **(B)** and RFBLUPS1416LOG0.09, Reproduction factor Log10 transformation for 2014 and 2016 BLUPs **(C)**. Genome-wise LOD threshold at 1% (P<0.01) and 5% (P<0.05) level of significance based on 1000 permutations above curves.

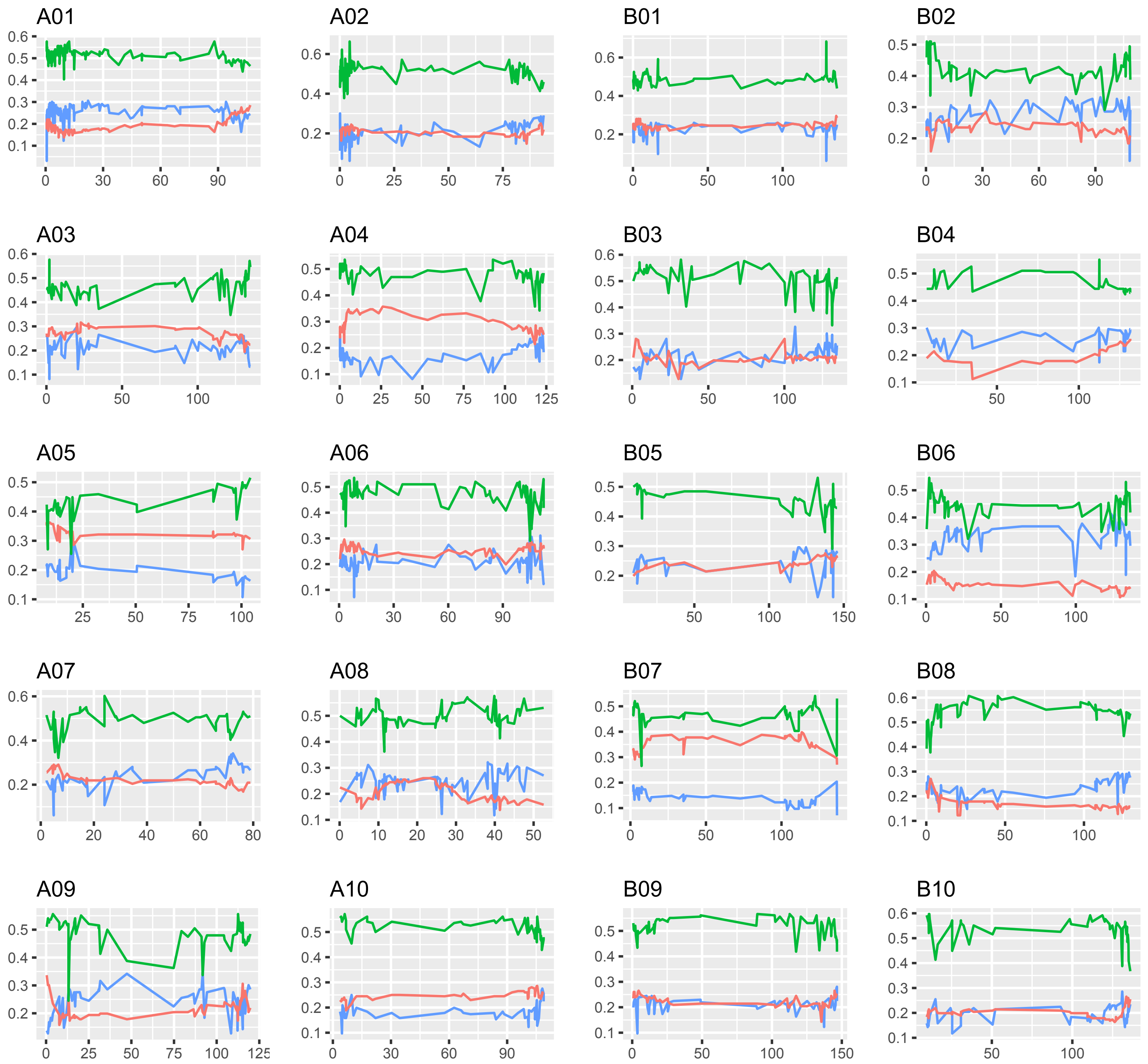

Figure S6: Distribution of segregation distortion across the 20 linkage groups in the RBS-F_2 population genetic map. Y-axis represents proportion of genotype (%) and X-axis represents physical position (Mbp). Wild alleles in blue, cultivated alleles in red and heterozygous alleles in green.
